# Perceptions of traditional Chinese medicine for chronic disease care and prevention: a cross-sectional study of Chinese hospital-based health care professionals

**DOI:** 10.1186/s12906-018-2273-y

**Published:** 2018-07-06

**Authors:** Xiaoqing Fan, Fanli Meng, Dahui Wang, Qing Guo, Zhuoyu Ji, Lei Yang, Atsushi Ogihara

**Affiliations:** 1 Medical School of Hangzhou Normal University, No. 16, Xuelin Street, Jianggan District, Hangzhou City, 310036 Zhejiang Province China; 2 Zhejiang Chinese Medical University, No. 548, Binwen Road, Binjiang District, Hangzhou City, 310053 Zhejiang Province China; 3 Faculty of Human Sciences, Waseda University, 1-104 Totsukamachi, Shinjuku-ku, Tokyo, 169-8050 Japan

**Keywords:** Traditional Chinese medicine, Chronic disease care and prevention, Chinese medicine hospitals, Health care professionals, Perception, Circumstance

## Abstract

**Background:**

In China, demands for disease prevention and health care and the prevalence of chronic non-communicable diseases have increased. TCM and general hospitals are increasingly utilizing TCM strategies for chronic non-communicable disease care and prevention. This study aimed to investigate health care professionals’ (HCPs’) perceptions of TCM for prevention, their TCM knowledge, and their abilities to provide such services in TCM and general hospitals.

**Methods:**

This cross-sectional study investigated Chinese medicine hospitals and Chinese medicine departments in general hospitals in five Chinese cities. A self-designed questionnaire used to study 400 HCPs focused on basic demographic data, the demand for and effects of TCM for prevention and treatment, and their perceptions of such service implementation. The data analysis included chi-squared tests and descriptive and multi-factor analyses.

**Results:**

The 335 HCP respondents comprised 230 (68.7%) females and 105 (31.3%) males, 75.5% of whom overall had knowledge of TCM preventive and health care services. Respondents older than 40 years (28.6%) had greater knowledge of and satisfaction with TCM for preventive and health care services than younger respondents. Moreover, 97.7% of the older respondents were clearly willing to provide TCM preventive services for chronic diseases, 67.8% of whom indicated that their hospitals already provided TCM for prevention and treatment. According to the chi-squared test results, the TCM service characteristics in hospitals, hospital outlooks regarding TCM and TCM development in hospitals were the primary factors affecting the respondents’ perceptions of TCM for chronic disease care and prevention. The multivariate analysis showed high satisfaction as significantly associated with older providers and those with lengthier work experience, particularly among those who worked in hospitals that provided typical TCM services and had positive attitudes towards TCM.

**Conclusion:**

The study HCPs had relatively satisfactory knowledge of and positive attitudes towards TCM for chronic disease care and prevention and would use it in practice. Their perceptions and satisfaction levels correlated closely with the successful application of TCM for preventive care and treatment in hospitals. While the use of TCM for prevention and treatment was well developed in some hospitals, further improvements are warranted.

## Background

Over thousands of years of practice, traditional Chinese medicine (TCM) has accumulated many methods, approaches, and beliefs for chronic disease care and prevention, which embody the philosophy of ‘Zhi-Wei-Bing’. Traditional Chinese medicine approaches to prevention and health care include emotional adjustment, seasonal health care, dietary care, herbal therapy, acupuncture, massage, detoxification, foot baths, etc. Traditional Chinese medicine is one type of traditional medicine that, as a complement and alternative to Western medicine, is widely accepted in China. Compared to Western medicine, traditional medicine has unique advantages, such as its safety, lower cost, effectiveness, and convenience, which leads to its high acceptance in an increasing number of countries [1, 2].

Thus far, the Chinese government has instituted nearly 100 regulations to support the development of TCM, particularly for chronic disease care and prevention. In December 2016, China introduced the first law on TCM [3]. Additionally, the Outline of the Strategic Plan for the Development of Traditional Chinese Medicine (2016–2030) and the Chinese Medicine Health Services Development Plan (2015–2020) detailed TCM service processes and standards for prevention and health care and directed hospitals and preventive health care institutions to implement the standards. Most regulations emphasized the importance of ‘Zhi-Wei-Bing’, which is crucial to realizing the unique role and advantages of TCM [4]. The concept of ‘Zhi-Wei-Bing’ is not only an important part of TCM but also a unique part of traditional Chinese culture. The ‘Zhi-Wei-Bing’ philosophy includes disease prevention, treatment, and rehabilitation, which is similar to the concept of prevention described by the World Health Organization (WHO) [5].

A literature review showed that chronic diseases, such as heart disease, stroke, cancer, diabetes and other chronic non-communicable diseases, resulted in a 63% mortality rate worldwide [2]. The prevalence of chronic disease is notably high in China, and continues to rise. The World Bank estimated that the cost of disease-related medical care (e.g., pertaining to cardiovascular diseases, diabetes, chronic obstructive pulmonary disease, etc.) will grow by nearly 50% in 2010–2030 in China [6]. The number of chronic disease patients, whose medical care costs account for 70% of the total disease cost burden in China, will increase to nearly 300 million [6]. Some studies have shown that one-third of human diseases might be avoided through prevention and appropriate disease management [2]. Thus, the prevention of chronic diseases is increasingly important.

An appropriate prevention and health care system would play a positive role in promoting the development of social economy. Many countries have established national preventive care and treatment systems, which have greatly improved their national health [7]. Although conventional health care has advanced worldwide, demonstrated by lower morbidity and mortality rates and improved life quality, complementary and alternative medicine (CAM) is widely used worldwide for preventive care and treatment services [8]. Global utilization of CAM is 9.8–76.0%, varying in the US, UK, Australia, and South Africa [5]. CAM services, such as herbal therapy, massage, acupuncture and moxibustion, and folk remedies, are similar in many countries [8].

Recently, the ‘Zhi-Wei-Bing’ philosophy has attracted the attention of numerous researchers in China and abroad, and many public health investigations are being performed in China. Most investigators are directing greater attention towards TCM preventive and health care services in the community rather than those by health care professionals in Chinese medicine hospitals.

Prevention and health care practitioners are the most active factors in traditional Chinese medicine hospitals, which are considered the principle facilities providing TCM preventive and health care services [2, 7]. This study aimed to investigate health care professionals’ attitudes, general practices, utilization willingness, and suggestions regarding the provision of TCM for prevention and health care in hospitals. This study also explored possible factors affecting health care professionals’ perceptions of and satisfaction with TCM for preventive and health care services. The findings will ideally provide guidance for medical professionals not only in China but also in other countries who seek to focus attention on chronic diseases and traditional medicine.

## Methods

### Data collections

Preventive health care professionals practice in two settings in urban China: independent health care workers work for centres for disease control and prevention and rehabilitation organizations, whereas in hospitals, preventive health care professionals provide part-time prevention and health care services in addition to disease treatment [6]. We chose health care professionals working in hospitals as the target respondents in this survey, including medical doctors, nurses, medical technicians, and in-hospital pharmacy staff. The reasons for this decision were that first, their work is particularly for ‘preventive purposes’. Second, they account for 63% of all preventive health care professionals. Last, the primary staff using TCM for chronic disease care and prevention are typically found in traditional Chinese medicine hospitals and traditional Chinese medicine departments in general hospitals. Though general hospitals mainly use allopathic Western medicine, their TCM departments typically use TCM products and technology for disease prevention and treatment, similar to TCM hospitals.

For the investigation, Beijing, Tianjin, Shanghai, Hangzhou, Guangzhou were chosen as research sites because they were the first to pilot the use of TCM for prevention and health care. Their developments of TCM for preventive and health care services were relatively favourable and representative. The five cities are distributed across the north, centre, and south of China, with similar economic levels, policy environments, and health services development. The research was reviewed and approved by the Ethics Committee of Hangzhou Normal University.

The survey was conducted from July to October 2015. One TCM hospital and one TCM department at a general hospital were selected from each of the five cities. The total sample size of 400 was estimated based on 15–20 times the number of questionnaire questions. According to the National Health and Family Planning Commission of the People’s Republic of China (PRC) and State Administration of Traditional Chinese Medicine (National Statistical Abstract of Chinese Medicine (2010–2015)), there were 646,000 TCM health care professionals in TCM hospitals and general hospitals in 2015. The number of TCM health care professionals differs between TCM hospitals and general hospitals, and approximately 48,000 TCM health care professionals work in TCM hospitals. The number of health care professionals providing TCM preventive and health care services in Chinese medicine hospitals is nearly 3 times the number in general hospitals. Therefore, we selected 60 individuals from TCM hospitals and 20 from general hospitals. The health care professionals included practising physicians (i.e., clinical, TCM and public health physicians), occupational assistant physicians, nurses, medical technicians and pharmacy staff.

### Measurement

The original questionnaire was designed based on the results of the literature review, group discussion and underwent several revisions according to feedback from health care professionals in TCM hospitals and general hospitals. This was aimed to ensure the description and wording was intelligible for all participants and improve the validity of the questionnaire. The pre-survey was conducted in Hangzhou, China, using a total of 80 questionnaires (response rate, 97%). Combined with the statistics from the State Administration of Traditional Chinese Medicine, the sample was representative. Based on the reflects from pre-survey, necessary modifications were made. Then a simple random sampling method was used. At the beginning, the random number table method was used to select five TCM hospitals and five general hospitals in the chosen cities. Then, the questionnaires were distributed to the chief hospital administrators by the State Administration of Traditional Chinese Medicine and were completed by health care professionals who provided TCM preventive and health care services. Finally, 400 health care professionals were surveyed; after eliminating incomplete and unclear questionnaires, the total number of responses was 373 (93.25%). After deleting administrative staff and support staff who were not health care professionals, 335 target respondents remained, yielding a response rate of 83.75%.

The information we collected encompassed basic demographic data, the perceptions of health care professionals and the current circumstances, and demands for and effects of TCM for prevention and health care in hospitals. Trained investigators (undergraduates and graduate students) collected the questionnaires immediately upon completion and determined whether any information was missing. When information was missing, the investigators contacted the respondents to modify the questionnaires. Trained investigators were also recruited to input the questionnaires. A random assessment was conducted before the data entry, and if the error rate exceeded 1%, re-entry was required.

### Statistical analysis

The database was established using Epidata 3.0, and the statistical analysis was performed using SPSS 20.0 for Windows. Categorical data were summarized as percentages, and continuous data were aggregated as the mean and standard deviation (SD). Initially, the demographic information of the respondents, their attitudes towards TCM, willingness to utilize TCM and TCM practice were analysed in a descriptive manner. Then, a chi-squared test was used to assess any possible relationships between the respondents’ answers (such as their attitudes, willingness and practice in hospitals) and their gender, age, education level, work experience, and practice of TCM for prevention and health care in hospitals. The level of statistical significance was defined as *P* < 0.05.

## Results

### Demographic characteristics of health care professionals

As shown in Table 1, most (230, 68.7%) of the 335 health care professionals surveyed were female. Most respondents were 20–39 years old (*n* = 135; 40.3%), and 28.6% (*n* = 96) were 40 years of age and older. The education level of the respondents was relatively high; 88.0% of the participants reported having undergraduate or higher education, whilst those with a college education or below were in the minority (*n* = 40; 12.0%). Most (*n* = 234; 69.8%) of the participants were practising physicians (i.e., TCM or clinical physicians). A small proportion (20%) of the respondents were chief or deputy chief physicians. Regarding the department in which they worked, 35.8% were in the internal medicine department and 23% were in the TCM department. Approximately half of the respondents had more than 6 years of work experience.

**Table 1:** Demographic characteristics of the health care professionals (*n* = 335)

Variables	Frequency (%)
Gender	
Male	105 (31.3)
Female	230 (68.7)
Age (years)	
20–29	135 (40.3)
30–39	104 (31.1)
40–49	52 (15.5)
50–59	34 (10.1)
≥ 60	10 (3.0)
Education	
Senior high school/secondary school	9 (2.7)
College	31 (9.3)
Undergraduate	118 (35.2)
Masters and above	177 (52.8)
Department	
Internal medicine	120 (35.8)
Surgery	19 (5.7)
Obstetrics and gynaecology	13 (3.9)
Paediatrics	13 (3.9)
Chinese medicine	77 (23.0)
Other departments	93 (27.8)
Position	
Practising physician (clinical)	66 (19.7)
Practising physician (TCM)	168 (50.1)
Practising physician (public health)	4 (1.2)
Occupational assistant physician	1 (0.3)
Nurse	60 (17.9)
Medical technician	27 (8.1)
Pharmacy staff	9 (2.7)
Title	
Chief physician	20 (6.0)
Deputy chief physician	47 (14.0)
Intermediate	107 (31.9)
Primary and below	161 (48.1)
Work experience	
≤ 1 year	65 (19.4)
2–5 years	82 (24.5)
6–10 years	69 (20.6)
11–15 years	41 (12.2)
> 15 years	78 (23.3)

**Table 2:** Health care professionals’ perception of TCM preventive and health care services

Items	N (%)
Familiarity	
Very familiar	56 (16.7)
Basic understanding	197 (58.8)
Unsure	49 (14.6)
Basically no understanding	28 (8.4)
Never heard of it	5 (1.5)
Advantages	
Prevention and control of complications	242 (72.2)
Few side effects	255 (76.1)
Good healing effect	189 (56.4)
High patient adherence	165 (49.3)
Low cost	130 (38.8)
Easy to use	121 (36.1)
Willingness to adopt	
Yes	166 (49.6)
Might try	161 (48.1)
No	8 (2.4)
Suitable method of promotion	
Educational presentation	255 (76.1)
TV & networking	241 (71.9)
Newspapers & books	223 (66.6)
Community promotion	211 (63.0)
Face-to-face discussion	213 (63.6)
Family & friends	117 (34.9)
Others	27 (8.1)

When analysing the relationships between the demographic characteristics and the health care professionals’ perceptions, we found that only position significantly impacted the medical staff’s perception (χ^2^ = 35.540, *P* < 0.01); moreover, practising TCM physicians had the deepest knowledge of TCM for prevention and health care (data table not shown).

### Health care professionals’ perception of TCM for prevention and health care

Table 2 presents among the 335 health care professionals surveyed, the knowledge rate of TCM for prevention and health care was 75.5%, including familiarity with or a basic understanding of TCM preventive and health care services. Concurrently, the knowledge rate of the group over 40 years of age was higher than that of the group younger than 40 years.

Compared with Western medicine, the advantages of TCM for prevention and health care were acknowledged. More than 50% of the respondents believed that the biggest advantages of TCM related to the prevention and control of complications, its few side effects and its satisfactory effects on healing. Overall, most (97.7%) health care professionals had used TCM for chronic disease prevention, and most respondents in this study believed that educational presentations, the use of electronic media and networking were effective methods to promote TCM for prevention and health care.

### Health care professionals’ practice of TCM for prevention and health care in hospitals

Table 3 shows that most (83.8%) of the health care professionals believed that TCM for prevention and health care was necessary for chronic disease prevention and control, whilst merely 3.3% considered it unnecessary. Moreover, 67.8% of the respondents believed that chronic disease care and prevention in their own hospitals already exhibited characteristics of TCM, though 23.0% were uncertain.

**Table 3:** Health care professionals’ prevention and treatment practices in hospitals

Items	N (%)
Requirements for implementation	
Completely needed	137 (40.9)
Basically needed	142 (42.4)
Uncertain	45 (13.4)
Basically not needed	9 (2.7)
Completely not needed	2 (0.6)
Incorporates elements of TCM	
Yes	227 (67.8)
No	31 (9.3)
Uncertain	77 (23.0)
Aspects for enhancement	
Bolster TCM training	251 (74.9)
Vigorously promote the role of TCM in chronic disease care and prevention	249 (74.3)
Enrich the content of TCM services	224 (66.9)
Increase the amount of TCM equipment	194 (57.9)
Increase TCM prevention and health care personnel	179 (53.4)
Lower fees	88 (26.3)
Improve service attitudes	80 (23.9)
Others	10 (3.0)

Health care professionals believed that an effective method to bolster the use of TCM in prevention and health care was to enhance Chinese medicine knowledge through education and training (74.9%) and to vigorously promote the role of TCM in chronic disease care and prevention (74.3%). In contrast, 23.9% of the health care professionals believed it necessary to improve their attitudes towards health care delivery.

### Circumstances and effects of TCM for prevention and treatment in hospitals

Table 4 shows that 86.3% of the respondents were supportive or completely supportive of using TCM methods of prevention and health care to prevent and control chronic diseases, whilst only 0.9% expressed that they were ‘not supportive’ of or ‘against’ such use.

**Table 4:** Circumstances and patient satisfaction regarding TCM for prevention and health care in hospitals

	N (%)
Health care professionals’ attitudes towards TCM for prevention and health care	
Very supportive	139 (41.5)
Supportive	150 (44.8)
Neutral	43 (12.8)
Not supportive	1 (0.3)
Against	2 (0.6)
Development of TCM for prevention and health care in hospitals	
Well developed	168 (50.1)
Some basic development but lacks advantages	153 (45.7)
Poorly developed	14 (4.2)
Patients’ satisfaction towards TCM prevention and health care	
Absolutely satisfied	32 (9.6)
Satisfied	174 (51.9)
Neutral	120 (35.8)
Not satisfied	8 (2.4)
Absolutely not satisfied	1 (0.3)

Health care professionals had different views on the current development of TCM for prevention and health care in hospitals: 50.1% believed that it was well-developed, 45.7% agreed that it has some fundamental development but lacks advantages, and only 4.2% believed the current situation was poorly developed.

Table 5 shows that 40% of the health care professionals were either satisfied or absolutely satisfied with the service diversity, charges, medical facilities and awareness of TCM for prevention and treatment provided by their hospitals. Nearly 50% of health care professionals had a neutral attitude towards TCM preventive health care service projects. Regarding their overall evaluation of TCM prevention and treatment, 50.4% of the health care professionals had a neutral attitude, whereas 44.2% were ‘satisfied’ or ‘absolutely satisfied’.

**Table 5:** Health care professionals’ satisfaction levels towards TCM for prevention and health care

Items (i.e., concerning TCM preventive health care service projects)	Satisfaction level				
	Absolutely not satisfied	Not satisfied	Neutral	Satisfied	Absolutely satisfied
Are you satisfied with the diversity of services?	5 (1.5%)	29 (8.7%)	162 (48.4%)	117 (34.9%)	22 (6.6%)
Are you satisfied with the charges?	6 (1.8%)	27 (8.1%)	175 (52.2%)	109 (32.5%)	18 (5.4%)
Are you satisfied with the medical facilities?	5 (1.5%)	34 (10.1%)	180 (53.7%)	99 (29.6%)	17 (5.1%)
Are you satisfied with the promotion methods?	7 (2.1%)	25 (7.5%)	157 (46.9%)	126 (37.6%)	20 (6.0%)
Are you satisfied with the overall project?	5 (1.5%)	13 (3.9%)	169 (50.4%)	126 (37.6%)	22 (6.6%)

### Analysis of the factors affecting health care professionals’ satisfaction

As demonstrated by the univariate analysis (Table 6), age and working experience (*P* < 0.05) were significantly associated with health care professionals’ overall satisfaction with TCM for prevention and treatment. Health care professionals 40 years of age and older or those who had more work experience were more likely to be satisfied with TCM for prevention and treatment in hospitals. Health care professionals 60 years of age and older had the highest overall satisfaction. Additionally, satisfactory development of TCM preventive and health care services, positive outlooks of hospitals towards TCM for prevention and treatment, and chronic disease prevention and treatment methods incorporating TCM characteristics were significantly associated with high satisfaction.

**Table 6:** Univariate analysis of different population characteristics and satisfaction with TCM for prevention and health care

	Items	Absolutely not satisfied (%)	Not satisfied (%)	Neutral (%)	Satisfied (%)	Absolutely satisfied (%)	χ^2^	P
Age (years)	20–29	1.9	3.1	47.5	38.1	9.4	40.85^a^	0.004^*^
	30–39	1.8	4.5	53.6	31.8	8.2		
	40–49	0.0	3.8	44.2	46.2	5.8		
	50–59	0.0	0.0	70.6	23.5	5.9		
	≥60	0.0	9.1	36.4	54.5	0.0		
Work experience (years)	≤1	1.4	5.2	42.9	42.9	7.5	28.607^a^	0.027^*^
	2–5	1.1	1.8	57.7	25.5	13.9		
	6–10	3.0	7.7	52.4	32.1	4.8		
	11–15	2.7	4.4	35.4	54.9	2.7		
	> 15	0.0	1.3	55.1	36.8	6.8		

*means statistically significan

**Table 7:** Logistic regression analysis of the factors affecting health care professionals’ overall satisfaction

Input variables	β	SD	Wald χ^2^	P	OR (95% CI)
Constant	−3.494	1.123	9.687	0.002	0.030
Development of TCM prevention and health care services in working hospitals	−0.009	0.431	0.000	0.983	0.991 (0.425, 2.307)
Attitude of working hospitals towards TCM for prevention and health care	−0.597	0.194	9.423	0.002^*^	0.551 (0.376, 0.806)
Whether chronic disease care and prevention in working hospitals has TCM characteristics	−1.400	0.284	24.317	0.000^*^	0.247 (0.141, 0.430)

*P < 0.05

Furthermore, the significant variables in the univariate analysis were used as independent variables, and all possible factors affecting health care professionals’ overall satisfaction were analysed using a step-wise logistic regression model. Finally, as Table 7 summarizes, two independent variables were entered into the regression equation: the outlook of hospitals towards TCM for prevention and treatment and whether the chronic disease care and prevention services in hospitals had TCM characteristics.

According to the factors affecting the overall satisfaction of the respondents in the logistic regression analysis, the outlook of hospitals towards TCM for prevention and treatment was a positive factor as was whether chronic disease care and prevention services in hospitals had TCM characteristics. Whilst other conditions remained unchanged, the worse the outlook of the hospital towards TCM prevention and treatment, the greater the possibility of low satisfaction (OR = 0.551, *P* < 0.01). Additionally, the results also showed that in hospitals where chronic disease care and prevention exhibited characteristics of TCM, health care professionals were more likely to be satisfied (OR = 0.247, *P* < 0.01).

## Discussion

This study investigated health care professionals’ perceptions of the current circumstances surrounding TCM for prevention and treatment in TCM hospitals and TCM departments in general hospitals. A total of 75.5% of the respondents had knowledge of TCM for chronic disease care and prevention, which approximated the fair knowledge (50–75%) reported by Suganya et al. in India [9]. To some extent, these results reflect the inadequate understanding of TCM for chronic disease care and prevention among health care professionals. At the same time, we can also see that HCPs with a better knowledge of TCM preventive and health care services are more likely to adopt and apply it in daily work. So it is necessary to improve their knowledge through more TCM training and medical practice. Also the more opportunities of applying TCM preventive health care technologies in the work, the more HCPs can understand the concept of TCM “Zhi-Wei-Bing”, recognize its necessity more, and have greater confidence in implementation.

Interestingly, older health care professionals (40 years of age and older) had better knowledge of TCM for prevention and treatment than younger health care professionals (40 years of age and younger). Though older individuals might become familiar with TCM at an earlier age than young individuals, because chronic diseases are more prevalent among older adults, the latter have greater requirements for preventive and therapeutic services [8]. Some surveys showed that health care professionals’ knowledge of TCM for prevention and health care were primarily obtained from government-issued literature [2]. Thus, internal promotional systems are an important method to improve their knowledge, along with publicizing the concept and philosophy of TCM. In our study, health care professionals acknowledged that educational presentations, the use of television and networking were more effective methods of disseminating TCM prevention and treatment information, whilst face-to-face discussions and recommendations from family and friends were less effective. These findings indicate that health care professionals are likely to trust official sources of information; therefore, government, social media and professional bodies might exert a positive effect on the further popularization of TCM for prevention and treatment.

Experts and researchers concluded that health care practitioners were optimistic about CAM; 67% would recommend its use and 63.3% believed that combination therapy using TCM and Western medicine was superior to Western medicine alone [8, 10, 11]. Medical personnel in Africa also had satisfactory experiences with CAM and identified an urgent need to include CAM in the health systems [12, 13]. The health care professionals in this study were positive about applying and constructing a prevention and health care system incorporating TCM characteristics. Most respondents were willing to utilize TCM measures for chronic disease care and prevention. This finding correlates with the results of another study in China, in which 86% of the medical personnel had positive attitudes towards TCM prevention and treatment [5]. Furthermore, most health care professionals agreed that hospitals should implement chronic disease care and prevention projects. Substantial data suggest that health care professionals would prefer to choose and incorporate CAM into their health services [14–17]. Perhaps due to the long history of TCM, its role has been widely confirmed [18]. The philosophy of ‘Zhi-Wei-Bing’ and its characteristics of simplicity, convenience, lower cost, and effectiveness in TCM have been accepted and promoted widely, and TCM treatment and health care services have been widely applied. Furthermore, perhaps the disadvantages of Western medicine in the treatment of chronic diseases have fuelled health care professionals’ optimism about TCM [19]. According to patients, CAM may not only cure [20] but also may counterbalance the adverse effects of Western medicine [21–23], improve physical and mental health, promote positive medical outcomes and wellness [24–27], etc. TCM may also enhance the comprehensive outcomes of preventive and treatment efforts and heighten the effectiveness of health interventions and assessments [28]. The conventional HCPs were ready to accept the idea of integrative medicine and the potential role of TCM products and practices in healthcare delivery [29]. However, practitioners’ understanding of CAM must be improved, with specific attention to issues of safety, regulation of evidence-based practice of CAM products and services in Ghana [29].

Approximately half of the health care professionals believed that current TCM preventive and health care services in the hospital had fundamental advantages, such as a favourable political congruence, professional personnel, broad trust among patients and others; however, some disadvantages remained. Some findings indicated that only 28.75% of practitioners believed that existing preventive and health care services demonstrated TCM characteristics, far less than those who believed that the services had no TCM characteristics (59%) [5, 6]. While despite the overall positive attitude towards CAM therapies, Ghanaian nurses do not perceive themselves to have sufficient knowledge of CAM [30]. To date, the Chinese government has introduced and implemented numerous policies and regulations throughout the country, particularly regarding TCM use in community health service centres. TCM for prevention and treatment is acknowledged and used increasingly by Chinese citizens. However, the use of conventional methods such as those of Western medicine remains the first and most common choice because of their excellent medical systems and rapid results [12, 31]. Fewer than half of the health care professionals were content with the current TCM preventive and health care services provided, and half reported having a neutral attitude. This finding indicates that the role of TCM in current prevention and health care remains unclear and that the role of TCM has not been fully explored.

The characteristics and advantages of TCM for prevention and treatment are powerful weapons for chronic disease care and prevention [32]. TCM hospitals and community health service institutions already have the capability to provide TCM prevention and health care services. However, they still need to diligently study and develop TCM prevention and health care service products, and to provide team building for medical personnel. The government must vigorously cultivate TCM HCPs, not only to increase training opportunities, but also to improve training contents, emphasize skills training, and set training content according to their work characteristics and work needs.The main skills of HCPs can include health status identification technology, health state intervention technology, Chinese medicine health assessment and physical fitness assessment, common prevention and rehabilitation programs, TCM preventive health care guidelines for high-risk groups of common diseases, and TCM health care technical operating norms.

A training system for HCPs in Chinese medicine should be established. It is necessary to require practitioners to have corresponding qualifications, such as qualifications of practicing physicians. Then a reasonable training plan should be established and training materials should be prepared. And combining job training with college education will make training more effectively.

According to the single factor analysis, position and work experience were significantly associated with health care professionals’ perceptions of TCM for prevention and health care services. Studies in Iran found that 88.4% of the participants had no previous complementary and traditional medicine education [17]. Therefore, the training of health care team members, particularly that of nurses and doctors, regarding the applications, benefits and side effects of complementary and traditional medicine is recommended [13]. To address the challenge of inadequate familiarity with and knowledge of TCM in China, continuing education in TCM is recommended for above-mentioned targeted health care professionals [33, 34]. Education for HCPs make us acknowledge HCPs’ beliefs strongly influence health teaching for patients and families. HCPs need to critically examine and reflect on the impact of culture, society and the media on their own health beliefs [35]. Additionally, reliable and accessible information concerning TCM for prevention and treatment should be made available in hospitals for both health care professionals and patients. In order to implement evidenced-based practice and teach in line with current evidence nurses need to critically examine and reflect on the impact of culture, society and the media on their own health beliefs [35]. Regarding the lack of work experience, organized education and training opportunities may address this challenge. The chi-squared test showed a significant association between older age and longer work experience with higher satisfaction with TCM among health care professionals. The enthusiasm of health care professionals directly affects their service attitude and quality; thus, the overall enhancement of TCM for prevention and treatment in hospitals is crucial.

Furthermore, hospitals’ outlooks on the application of TCM for chronic disease care and prevention and whether the services reflect TCM characteristics might contribute to differences in the perspectives and satisfaction levels of health care professionals. Overall, we suggest the improved development of TCM preventive and health care services, positive outlooks of hospitals towards TCM for prevention and treatment, and the incorporation of TCM-based methods into hospitals’ practices concerning chronic disease care and prevention. Hospitals should strengthen the training of high-level TCM HCPs, improve the reward mechanism, and prevent the outflow of talents. As hospitals become more proactive regarding TCM practice for prevention and treatment, health care professionals will be more likely to have better knowledge of and higher satisfaction with TCM. Traditional Chinese medicine preventive and health care services should be rooted in TCM hospitals and general hospitals as centres of care, and aligned with community health service centres.

This study has some limitations. The first is related to the background setting and small sample size. When the questionnaires were distributed, some administrative and support staff surveys were admixed into the survey, which led to smaller matched samples. The five selected cities were TCM pilot locations, and their TCM development was relatively advanced; thus, the research results were not representative of the whole country. Second, the data were gathered from health care professionals’ self-reporting regarding their TCM awareness and practices, without spot visits and objective verification. To the best of our knowledge, this study was one of the few domestic projects to evaluate current TCM for chronic disease care and prevention from the perspective of health care professionals in hospitals rather than communities, which might be a starting point for similar follow-up studies. The evaluation of the specific satisfaction and outcome indicators of TCM for prevention and treatment is necessary.

## Conclusion

In this study, we surveyed hospital-based health care professionals concerning TCM for prevention and treatment. The respondents had relatively satisfactory knowledge of and positive attitudes towards TCM for prevention and confirmed its contribution to chronic disease care and prevention. Moreover, their perceptions and satisfaction levels were closely related to the application of TCM for prevention and treatment. Currently, the use of TCM for prevention and treatment has been well developed in some hospitals, though further improvement is needed. To enhance the use of TCM for prevention and treatment, professional training of medical personnel must be provided along with enriched service contents and service levels. This study also provides guidance for establishing chronic disease care and prevention service programs.

## Abbreviations

CAM: Complementary and Alternative Medicine
HCP: Health care professional
TCM: Traditional Chinese Medicine
WHO: World Health Organization
