# Correction to: Perceptions of traditional Chinese medicine for chronic disease care and prevention: a cross-sectional study of Chinese hospital-based health care professionals

**DOI:** 10.1186/s12906-019-2434-7

**Published:** 2019-01-22

**Authors:** Xiaoqing Fan, Fanli Meng, Dahui Wang, Qing Guo, Zhuoyu Ji, Lei Yang, Atsushi Ogihara

**Affiliations:** 1 Medical School of Hangzhou Normal University, No. 16, Xuelin Street, Jianggan District, Hangzhou City, 310036 Zhejiang Province China; 2 Zhejiang Chinese Medical University, No. 548, Binwen Road, Binjiang District, Hangzhou City, 310053 Zhejiang Province China; 3 Faculty of Human Sciences, Waseda University, 1-104 Totsukamachi, Shinjuku-ku, Tokyo, 169-8050 Japan

**Correction to: BMC Complement Altern Med (2018) 18:209**

**https://doi.org/10.1186/s12906-018-2273-y**

Following publication of the original article [1], the author reported that the funding information was missing from the original article.

The funding information is as follows:

This study was supported by the National Natural Science Foundation of China (No.71603068). The project is online and offline health management service path of middle-aged and young people’s hypertension in community:a research based on process reengineering.
